# Gestational arsenic exposure induces anxiety-like behaviors in F1 female mice by dysregulation of neurological and immunological markers

**DOI:** 10.1265/ehpm.23-00046

**Published:** 2023-07-05

**Authors:** Chaw Kyi-Tha-Thu, Soe-Minn Htway, Takehiro Suzuki, Keiko Nohara, Tin-Tin Win-Shwe

**Affiliations:** 1 Department of Immunology, International University of Health and Welfare School of Medicine, Narita Campus, Chiba, Japan; 2 Department of Physiology, University of Medicine, Magway, Magway, Myanmar; 3 Health and Environmental Risk Division, National Institute for Environmental Studies, Tsukuba, Japan

**Keywords:** Anxiety, Arsenic, Gestational exposure, F1 female mice, Neurotransmitter, Apoptosis

## Abstract

**Background:**

Arsenic is a harmful heavy metal and a well-known developmental neurotoxicant. Previously, we have reported that gestational arsenic exposure resulted in impaired social behaviors in F1 and F2 male mice. However, little is known about the developmental arsenic exposure on anxiety-like behavior. This study aimed to detect the effect of gestational arsenic exposure on anxiety-like behavior and related gene expressions in 74-week-old F1 female mice.

**Method:**

Pregnant C3H/HeN mice (F0) were given drinking water containing 85 ppm sodium arsenite (NaAsO_2_) from gestational day 8 to 18. The control mice were given tap water only. At 74-week-old, open field test was performed, then anxiety and apoptosis-related factors were determined by real_time RT_PCR and immunohistochemical analyses.

**Results:**

The arsenite-exposed F1 female mice showed decreased center entry and center time in open field test. In addition, the number of grooming and fecal pallet was significantly increased in the arsenite-exposed F1 female mice compared to the control. Downregulation of brain-derived neurotrophic factor (BDNF), serotonin receptor (5HT1A) and upregulation of nuclear factor kappa-light-chain-enhancer of activated B cells (NFκB), interleukin 1 β (IL-1β), cyclooxygenase 2 (COX2), caspase-3, Bcl2-associated X protein (Bax) were detected in the prefrontal cortex in the arsenite-exposed F1 female mice. Microglial marker ionized calcium-binding adapter molecule 1 (Iba1)-positive cells were increased in the arsenite-exposed F1 female mice. Moreover, a significantly increased plasma corticosterone level was observed in the arsenic-exposed F1 female mice.

**Conclusions:**

This study suggested that gestational arsenic exposure induced anxiety-like behavior accompanied with dysregulation of neurological and immunological markers, neuroinflammatory responses, neuronal apoptosis, and decreased neurogenesis in the prefrontal cortex of F1 female mice.

## Background

Heavy metals, metallic elements, are persistent environmental pollutants and have been reported as environmental risk factors for multi-organ dysfunction. Parallel with rapid industrialization and urbanization, human exposure to heavy metals have increased globally [1, 2]. Humans are exposed to heavy metals generally via food, water, and air [3]. Due to their potential adverse effects on human health, heavy metal exposures have become a major public health issue [4]. Previously, it has been demonstrated that environmental heavy metals may be risk factors of mental illness, especially depression [5–7]. However, little is known about the association existing between heavy metal exposure and anxiety.

Anxiety means excessive worrying which is characterized by restlessness, irritability, fatigue and difficulty in concentration, and it can affect daily life in workplaces, school environment and society [8]. Among the heavy metals, lead, arsenic, mercury and cadmium are well known neurotoxicants and exposure to these metals cause IQ decrements, learning and memory impairment, however, few studies have shown that the link between metal exposure and anxiety. Regarding human studies, it has been reported that association between lead exposure and anxiety-like behaviors in childhood and adults [9–11]. It was also demonstrated that relationship between manganese exposure and anxiety-like behaviors in adults [12, 13]. In animal studies, long-term cadmium exposure induced anxiety-like behaviors in male and female rats [14]. Recently, it was reported that gestational arsenic exposure may induce anxiety-like behaviors in adult offspring via DNA methylation changes in fetal brain [15].

Arsenic is a well-known environmental toxicant to various human organs including brain. Arsenic contamination of ground water is the global health concern, particularly more than half of the world population is affected [16]. Many studies reported that prolong exposure of high level of arsenic in drinking water causes arsenicosis with various health complications such as skin lesions and cancer. Recently, scientists have been explored the possible therapy to lessen the arsenic toxicity in the community such as techniques of arsenic removal from water [17, 18] and investigation of therapeutic candidate compound for arsenic-induced DNA damage [19, 20]. Retrospective studies reported that arsenic-contaminated drinking water exposure during perinatal period showed impairment of cognitive abilities in the childhood [21, 22]. This strongly indicated that arsenic could cross the placenta barrier, reach to the brain through the blood brain barrier, accumulate in different parts of the brain leading to the tissue damage or DNA damage and finally give adverse effects to the neurobehavioral development. However, the effect of arsenic exposure during gestational period on anxiety-like behavior in the F1 offspring is still unclear.

Acute or chronic exposure of environmental toxins causes the abnormal biological responses including neuroinflammation and psycho-social impairment including depression and anxiety. All anxiety disorders are highly related to both physical and mental wellbeing. Anxiety is an uncomfortable feeling of our body’s natural response to stress or uncertain condition. In mammalian species, prefrontal cortex (PFC) regulates the anxiety like behaviors such as restlessness, increased heart rate and autonomic functions, agoraphobia, self-isolation, decrease ability to perform daily activities in human [23]. In rodents, anxiety-like behaviors can be examined by open field test, novelty-induced recognition test, social interaction test and elevated plus-maze test [24–26]. Early life stress induces anxiety-like behaviors were occurred by dysregulation of neuronal plasticity in PFC to amygdala circuits [27]. Maternal exposure to heavy metals is remarkably associated with pediatric morbidity including preterm births, malformations, pulmonary, cardiovascular, and metabolic diseases, and behavioral problems [28]. Recently, the Japan Environment and Children’s Study group has reported that association between paternal occupational exposure including heavy metals like chromium, arsenic, cadmium and the risk of infant congenital heart defects [29]. Drinking water contamination of arsenic can be considered as an environmental stress to the developing brain. A study reported that accumulation of arsenic in the brain is more prominent in F1 adult offspring than in F0, consequently resulting in more significant neurobehavioral changes [30]. Moreover, gestational arsenic exposure showed sex-dependent neurotoxicity due to the resistance of epigenetic reprogramming of the glucocorticoid signaling system [31, 32]. In our previous report, we showed that gestational arsenic exposure causes the neurotoxicity and social behavior impairment in the F1 and F2 male mice [33, 34].

Anxiety is the common mental disorder and women are significantly vulnerable to develop an anxiety disorder than men throughout the lifespan [35, 36]. According to the National Comorbidity Survey, prevalence rates for anxiety disorder were 30.5% for women and 19.2% for men [37]. The hippocampus, amygdala and PFC are the major brain areas which control emotional behaviors and functions. The neural connections projecting from ventral hippocampus to the PFC are unidirectional and ventral hippocampus also has bidirectional connections with the amygdala, and the amygdala has bidirectional connections with the PFC. Therefore, we aimed to examine the gestational arsenic exposure on anxiety-like behavior and neurological and immunological markers in the PFC in the later life of F1 female offspring.

## Methods

### Animals and arsenic exposure

**Fig. 1:**
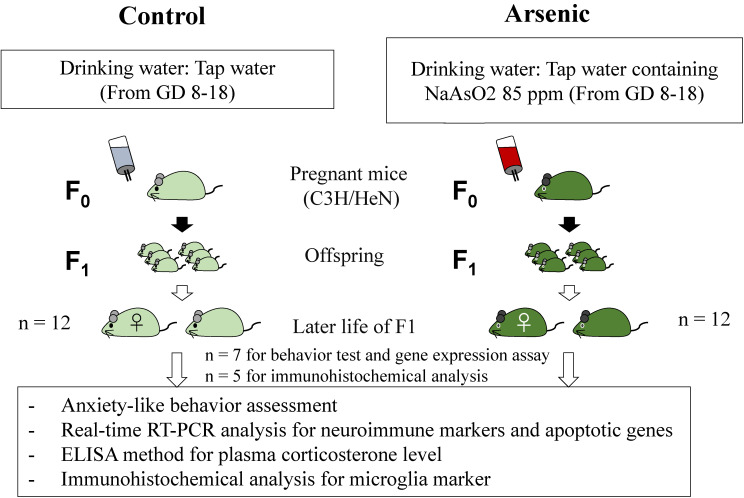
Experimental design. The C3H/HeN pregnant mice (n = 14) were given arsenic (NaAsO2, 85 ppm (85 mg/L)) containing tap water or only tap water during the 8–18 days of the gestational period. From the offspring, the F1 female mice (n = 7) were randomly selected and fostered for the experiments. At the age of 74-week-old, these mice were used for behavior assessment and molecular analyses.

Pregnant C3H/HeN mice (F0, total n = 14) were purchased from JAPAN SLC (Shizuoka, Japan) and these mice (n = 7) were given arsenic exposure (sodium arsenite, NaAsO2, 85 ppm (85 mg/L)) in the drinking water starting from the gestational day (GD) 8 to 18. Control mice (n = 7) were given tap water only. The experimental dose for gestational arsenic exposure was explained in our previous studies [33, 34, 38]. The experimental design of this study was illustrated in the Fig. 1. F1 female offspring (n = 12 per each group) delivered from arsenic exposed F0 or control F0 were randomly collected for the experiments. At the age of 74-week, the control female mice (*n* = 7) and arsenic-exposed female mice (*n* = 7) were used for anxiety-related behavioral test and molecular analyses. Then, the control F1 female mice (*n* = 5) and arsenite F1 female mice (*n* = 5) were used for immunohistochemical analyses. This study was approved by the Ethics Committee of the Animal Care and Experimentation Council of the National Institute for Environmental Studies (NIES), Japan (Ethics approval code number: AE-22-12).

### Open field test

The 74-week-old arsenic-exposed and control F1 female mice were examined for anxiety-like behaviors using open field apparatus (Muromachi Kikai Co. Ltd., Tokyo, Japan). A transparent squared chamber (100 cm × 100 cm) with 35 cm depth was provided. A center square (25 cm × 25 cm) was marked with red tape inside the chamber. An experimental mouse was placed in the center of the chamber and allowed to explore it for 5 min. The behaviors of the individual mouse were recorded by a video tracking system with ANY-maze software (Stoelting Co., Wood Dale, IL, USA) and subsequently performed anxiety-like behavior analyses such as monitoring the latency to enter into the center, rearing (standing on the hind limbs) and grooming (washing the face and body repeatedly) behaviors, counting the number of entries into the center and defecation (counting fecal pallets), measuring the time spent in the center.

### Plasma corticosterone ELISA

Blood samples were taken by cardiac puncture and put into heparinized tubes. The heparinized blood samples were centrifuged (3000 rpm for 15 min at 4 °C) to isolate plasma and stored at −20 °C until analysis. Plasma corticosterone levels were quantified with an enzyme-linked immunosorbent assay (ELISA) kit (EIA-4164; DRG Diagnostics, Marburg, Germany) in accordance with the manufacturer’s instructions.

### Messenger RNA expression assay

The prefrontal cortex (PFC) was collected from each mouse of the control and arsenite exposed 74-week-old F1 female mice (n = 7 per each group) and was frozen in liquid nitrogen and then stored at −80 °C. Total RNA extraction was performed by EZ-1 RNA tissue mini kits and BioRobot EZ-1 (Qiagen GmbH, Hilden, Germany). Purity and concentration of total RNA was examined by ND-1000 NanoDrop RNA Assay protocol (NanoDrop Technologies, Wilmington, DE, USA). Then, first strand cDNA synthesis from total RNA was done by SuperScript RNase H-Reverse Transcriptase II (Invitrogen, Carlsbad, CA, USA) and thermal cycler (Gene Atlas E, ASTEC, Fukuoka, Japan). The mRNA expression levels of 18S rRNA (internal control), NFκB (nuclear factor kappa B), IL-1β (interleukin 1β), COX2 (cyclooxygenase 2), Bax (nuclear factor kappa-light-chain-enhancer of activated B cells), Caspase-3, 5HT1A (5-hydroxytryptamine (serotonin) receptor 1A), Drd2 (dopamine receptor D2) and BDNF (brain-derived neurotrophic factor) were determined by using Light Cycler 96, Roche, Germany. The primer sequences used for neurological and immunological markers were expressed in Table 1. Data were analysed by using comparative threshold cycle method. The relative mRNA expression levels were expressed as mRNA signals per unit of 18S rRNA expression.

**Table 1:** Primers sequences used for gene expression assay.

**Primer name**	**Primer sequence (5′ to 3′)**
NFκB	Forward: GCTGCCAAAGAAGGACACGACA
	Reverse: GGCAGGCTATTGCTCATCACAG
IL-1β	Forward: TGGACCTTCCAGGATGAGGACA
	Reverse: GTTCATCTCGGAGCCTGTAGTG
COX2	Forward: GCGACATACTCAAGCAGGAGCA
	Reverse: AGTGGTAACCGCTCAGGTGTTG
Bax	Forward: TCAGGATGCGTCCACCAAGAAG
	Reverse: TGTGTCCACGGCGGCAATCATC
Caspase-3	Forward: GTGGAACTGACGATGATATGGC
	Reverse: CGCAAAGTGACTGGATGAACC
5HT1A	Forward: CTGGGGACGCTCATTTTCT
	Reverse: CCAAGGAGCCGATGAGATAG
BDNF	Forward: CTGACACTTTTGAGCACGTGATC
	Reverse: AGGCTCCAAAGGCACTTGACT
Drd2	Forward: TGTACAATACGCGCTACAGCTCCA
	Reverse: ATGCACTGCGTTCTGGTCTGCGTTA
18S	Forward: TACCACATCCAAAAGGCAG
	Reverse: TGCCCTCCAATGGATCCTC

### Immunohistochemical analysis

Microglia are one of the immune cells in the brain, and microglial activation indicates neuroinflammation and neurotoxicity. To detect microglial activation, the brain sections were immunostained with microglial marker *Iba1* as described previously [39]. Briefly, the brain sections were immersed in absolute ethanol followed by 10% H_2_O_2_ for 10 min each at room temperature. After rinsing in 0.01-M phosphate buffer saline, the sections were blocked with 2% normal swine serum in PBS for 30 min at room temperature and then reacted with goat polyclonal anti-Iba1 (diluted 1:100; abcam: ab5076; Tokyo, Japan) in PBS for 1 h at 37 °C. Then, the sections were reacted with biotinylated donkey anti-rabbit IgG (1:300 Histofine; Nichirei Bioscience, Tokyo, Japan) in PBS for 1 h at 37 °C. The sections were then incubated with peroxidase-tagged streptavidin (1:300, ABC KIT) containing PBS for 1 h at room temperature. After a further rinsed in PBS, Iba1 immunoreactivity was detected using a Dako DAB Plus Liquid System (Dako Corp., Carpinteria, CA, USA). To detect Iba1-positive microglia, photomicrographic digital images (150 dpi, 256 scales) of the PFC region were taken using a CCD camera connected to a light microscope. Numbers of Iba1-positive microglia in the PFC were quantified in high power field using ImageJ software (5 fields per section and 3 sections per mouse, n = 5 mice/group).

### Statistical analysis

All the data were presented as mean ± standard error (SE). Statistical Package for the Social Sciences (SPSS)-version 26 (IBM Corp., Armonk, NY) was used for statistical analysis. To detect the body weight, brain weight, behavioral performance, plasma corticosterone level, the messenger RNA expression levels and Iba1 positive cell, Student’s *t* test was used to analyze between the control and arsenite groups. Difference at *p* < 0.05 was regarded as significant.

## Results

### Assessment of general toxicity

**Fig. 2:**
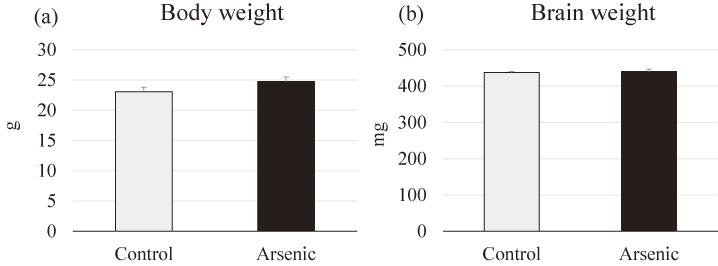
Measurement of body weight and brain weight. (a) Body weight and (b) brain weight of the control and the arsenic-exposed 74-week-old F1 female mice offspring (n = 7 for each group).

To access the generalized toxicity after gestational arsenic exposure, we compared the body weight and brain weight of the control and arsenite group of the F1 female offspring. There were no significant differences in both body weight and brain weight of gestationally arsenic exposed F1 female offspring compared to the control group (Fig. 2).

### Assessment of anxiety-like behavior

#### Open field test

**Fig. 3:**
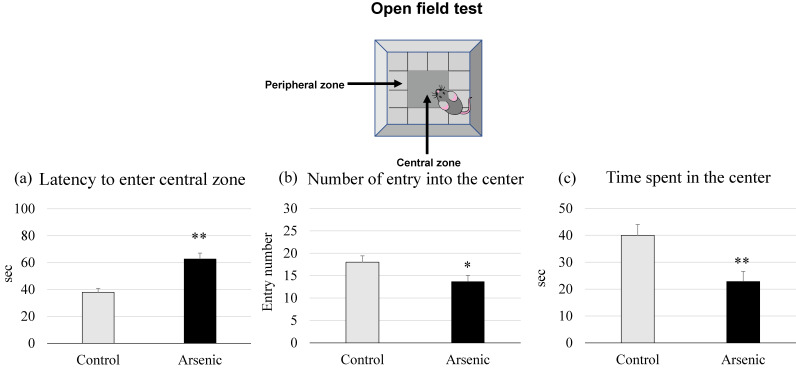
Effect of gestational arsenic exposure on anxiety-like behavior: Open field test. Analysis of (a) the latency to enter into the center, (b) the number of entry into the central zone and (c) the time spent in the center in the control and the arsenic-exposed 74-week-old F1 female mice offspring. Each bar represents the mean ± SE (n = 7 per each group, *p < 0.05, **p < 0.01).

Open field test was used to asess the anxiety-like behaviors. Gestational arsenic-exposed F1 female offspring showed a significant latency to enter into the center and a significant fewer entry number compared to the control group (*p* < 0.05, Fig. 3a and b). The time spent in the central zone was significantly shorter duration in the arsenic-exposed F1 female group than the control (*p* < 0.05, *p* < 0.01, Fig. 3c). Our findings indicate that arsenic exposure during gestational period may induce the anxiety-related behaviors in the later life of F1 female mice.

#### Behaviors during open field test

**Fig. 4:**
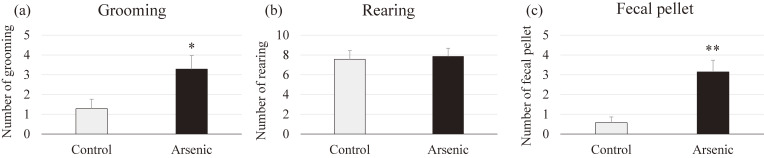
Assessment of restlessness and anxiety-related behavior during open field test. (a) The number of rearing, (b) grooming and (c) the number of fecal pellet in the cage of the control group and the arsenic group of 74-week-old F1 female mice. Each bar represents the mean ± SE (n = 7 per each group, *p < 0.05, **p < 0.01).

We also examined additional behavior to confirm the anxiety in arsenic-exposed mice. The repetitive behaviors of grooming and rearing in mice indicate the restlessness and the novelty stress induces more fecal pellet output. The number of grooming behavior in the arsenic-exposed mice was significantly increased when compared to that of control group (*p* < 0.05, Fig. 4a), whereas there was no difference in number of rearing behavior among groups (Fig. 4b). Moreover, the number of fecal pallet output was significantly increased in the arsenic-exposed mice suggesting that arsenic-exposed brain may have high potential of lower stress resistance level than the control brain (*p* < 0.01, Fig. 4c).

### Plasma corticosterone level

**Fig. 5:**
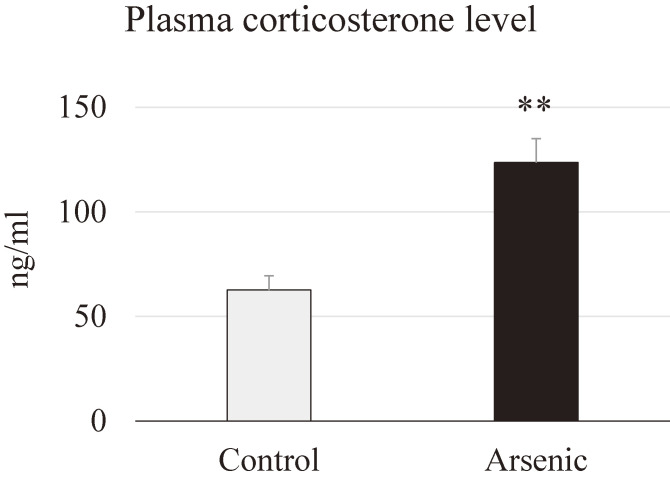
Plasma corticosterone level. Corticosterone concentration in plasma of 74-week-old female mice with or without gestational arsenic exposure. Each bar represents the mean ± SE (n = 7 per each group, **p < 0.01).

Plasma corticosterone level was analyzed in the 74-week-old female mice with or without gestational arsenic exposure to examine the hyperactivity of the hypothalmus-pituitary-adrenal (HPA) axis. A significant elevation of corticosterone level was observed in the plasma of arsenic-exposed F1 female mice. This data indicates that gestational arsenic exposure group is very sensitive to stressor like environmental chemical exposure (Fig. 5).

### Expression of messenger RNA level in the prefrontal cortex

**Fig. 6:**
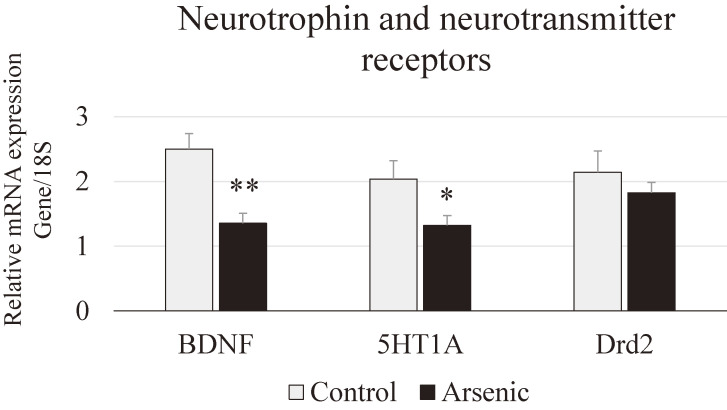
Messenger RNA expression level of neurotrophin and neurotransmitter receptors. Neurotrohphin BDNF and, neurotransmitter receptors 5HT1A, and Drd2 mRNAs in the PFC of the control group and the arsenic group of 74-week-old F1 female mice. Each bar represents the mean ± SE (n = 7 per each group, *p < 0.05, **p < 0.01).

Neurotrophin and neurotransmitter receptors: Neurotrophic factor such as brain-derived neurotrophic factor (BDNF) and anxiety-related neurotransmitters such as serotonin receptor 5HT1A, dopamine receptor Drd2 in the PFC were examined. BDNF and 5HT1A mRNAs were significantly decreased in arsenic-exposed group compared to the control group (*p* < 0.05, *p* < 0.01, Fig. 6). These findings indicate that gestational exposure to arsenic may induce reduction of neurogenesis and impaired neurotransmission.

**Fig. 7:**
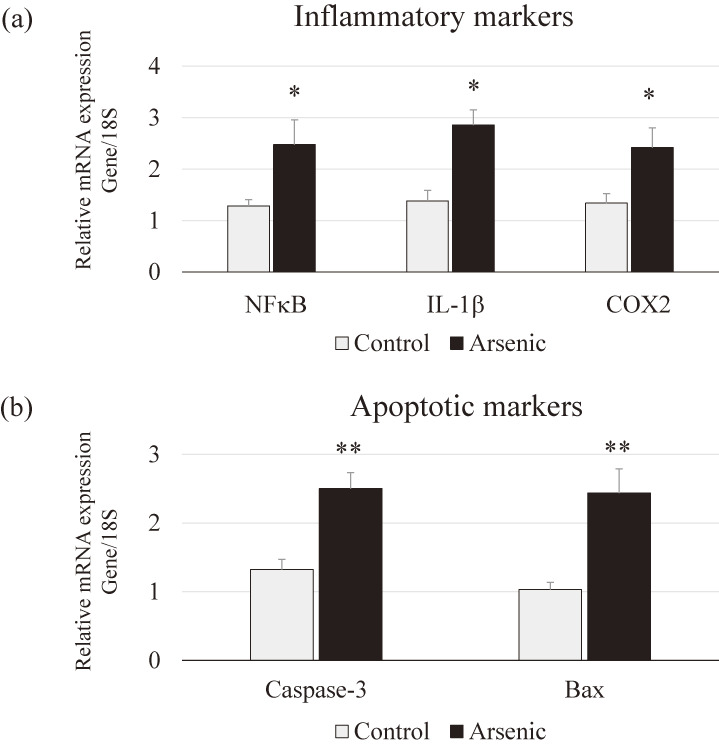
Messenger RNA expression level of inflammatory and apoptotic markers in the PFC. (a) Inflammatory markers NFκB, IL-1β, and COX2, (b) apoptotic markers caspase-3, and Bax mRNAs in the PFC of the control group and the arsenic group of 74-week-old F1 female mice. Each bar represents the mean ± SE (n = 7 per each group, *p < 0.05, **p < 0.01).

Inflammatory and apoptotic markers: To detect arsenic-induced neuroinflammation in the PFC, the inflammatory molecular markers such as NFκB, IL-1β, COX2, and apoptosis markers such as caspase-3 and Bax in the PFC were examined. Inflammatory and apoptotic markers mRNAs were significantly increased in the arsenic-exposed group compared with the control group (p < 0.05, *p* < 0.01; Fig. 7). These results indicate that gestational exposure to arsenic may induce inflammatory response as well as apoptosis in 74-week-old F1 female mice.

### Immunohistochemical analysis

**Fig. 8:**
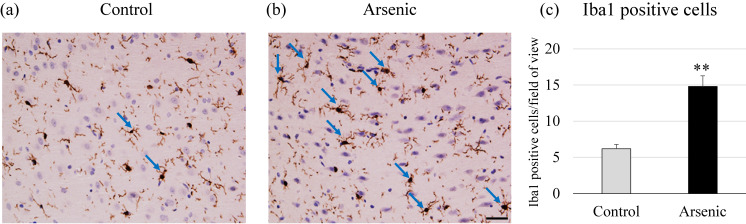
Detection of activated microglia in the PFC. Representative photomicrographs of activated microglia in (a, left panel) the control and (a, right panel) arsenic group of 74-week-old F1 female mice, and (b) the number of microglia marker Iba1 positive cells in the prefrontal cortex. Scale bar = 50 µm. Each bar represents the mean ± SE (n = 5 per each group, **p < 0.01). Blue arrow indicates activated microglia.

Activation of microglia in the PFC was examined using microglial marker *Iba1*. Representative digital photomicrographs of *Iba1*-immunostained brain sections taken from the PFC of the control and arsenic-exposed groups were shown in Fig. 8a. Microglial activation was markedly increased in the PFC of the arsenic-exposed group as compared with that in the control group *Iba1*-positive microglia in the PFC were quantified under a high-power field using ImageJ software (Fig. 8b).

## Discussion

Major finding of this study was that arsenic exposure in the gestational period caused long-term adverse effects in the 74-week-old female mice. First, gestational arsenic exposure induced neuropsychological behavioral changes mainly anxiety-like behaviors such as delayed entry time into the center, decreased number of entries into the center, shorten time spending in the center in the arsenic-exposed female mice. In addition, other anxiety-related symptoms such as grooming, and defecation were remarkably increased in the arsenic-exposed female mice. Second, reduction of neurogenesis- and anxiety-related neurotransmitters such as BDNF, 5HT1A and Drd2 in the PFC was observed. Third, increased neuroinflammatory markers (NFkB, IL-1β, COX2) and apoptotic markers (caspase-3 and Bax) in the PFC were occurred in the later life of gestationally arsenic-exposed female offspring. The possible mechanism of arsenic-induced neurotoxicity may be related to the dysregulation of neurological and immunological markers resulting in the reduction of neurogenesis, impaired anxiety-related neurotransmitter functions as well as neuroinflammation and apoptosis which in turn may induce anxiety-like behaviors in arsenic-exposed female mice.

Our series of experimental design based on the Waalkes and colleagues who have reported that among the different mouse strains, pregnant C3H/HeNCr (C3H) mice receiving sodium arsenite (NaAsO_2_) in the drinking water at 85 ppm (100 ppm showed maternal toxicity and 42.5 ppm showed very low carcinogenicity) from day 8 of gestation through to delivery was suitable schedule for transplacental toxicological assessment of carcinogenicity [40]. The current WHO recommended limit of arsenic in drinking-water is 10 µg/L (0.01 ppm), while millions of people around the world are exposed to arsenic at concentrations much higher than the guideline value (100 µg/L or greater) [41]. The reason for high doses setting is lower susceptibility of mice to arsenic [42]. As mentioned above, in our previous and present studies, NaAsO_2_, 85 ppm was given to F0 pregnant mice from gestational days 8–18 to detect carcinogenicity, reproductive and neurotoxicity in F1 and F2 offspring [33, 34, 43–45]. This is a standard dose to detect the effects of gestational exposure to arsenic without maternal toxicity and teratogenicity.

Exposure to the environmental chemicals during developing period can lead to permanent health hazards, particularly irreversible neurotoxicity and brain damage. Even low dose exposure can induce adverse effects with the slow onset. Low dose arsenic exposure in the embryonic zebra fish induced persistent morphological and locomotor deficits during juvenile to adulthood, but no anxiety-related behaviors were observed in the arsenic-exposed adult zebra fish [46]. Developmental arsenic exposure of newborns via contaminated milk power showed permanent changes in neurological and neuropsychological behaviors in elderly life [47, 48]. In our previous report, we observed that gestational arsenic exposure showed poor social behavior in the 74-week-old male mice of both F1 and F2 offspring [33, 34]. Actually, in this study, we designed our protocol to examine the social behavior and anxiety-related behavior in arsenic-exposed F1 female mice. We had a plan to compare social behavior of F1 female mice to our previous data of F1 male mice. Unfortunately, software system for social behavior test was broken down and because of study age limitation, we have examined only anxiety-related behaviors in F1 female mice in the present study. The aim of this study is to investigate the effect of gestational arsenic exposure on anxiety-like behaviors and anxiety-related gene expression in the PFC of 74-week-old F1 female mice. In this study, we focused on anxiety in the female mice because the retrograde epidemiological study showed that women have higher rate of incidence and prevalence for anxiety disorders than men [49]. Anxiety disorders become a high concern in global health due to their high prevalence and incidence, lifelong impairment, and disturbance of the normal daily activity. The level of anxiety is not dependent to the age, but the occurrence of anxiety is more in elderly people compared to the young teenagers. Anxiety become more common in middle-aged to old-aged people probably due to the increasing exposure to various stressful conditions including environmental toxicants.

BDNF is a neurotrophin, which is abundantly expressed in the brain, and it is also present in the blood. BDNF is regarded to be negatively related with the neuropsychiatric disorders such as cognitive malfunction, anxiety and depression [50–52]. Regarding environmental heavy metal exposure, our previous study has shown that developmental arsenic exposure induced poor sociability and social novelty preference accompanied with decreased BDNF mRNA expression in the prefrontal cortex of mice [33]. A cross-sectional study recently reported that reduction of cognitive function together with low level of serum BDNF was found in the Bangladesh people using arsenic-concentrated water [53]. Moreover, it has been reported that inverse relationship between manganese exposure and plasma BDNF levels in occupational exposed workers [54]. Zhang et al., (2020) reported that early-life stress triggered by prolong maternal separation showed reduction of BDNF protein in the PFC of rat brain, reduction of plasma corticosterone as well as reduction of brain weight and locomotor function in the prolong maternal separation rats compared to that of control rats [55]. In this study, we found that plasma corticosterone level and BDNF gene expression in the PFC was significantly decreased in the gestationally arsenic-exposed F1 female mice, which is also correlated with anxiety-like behaviors in those mice. Our findings highly suggested that gestational arsenic exposure induced anxiety-related disorders by regulating BDNF signaling neural pathway. As for the further investigation the detailed mechanisms of anxiety-like behavior accompanied with downregulation of brain-derived neurotrophic factor in F1 female mice should be elucidated.

The HPA axis and the serotonergic neuromodulatory system plays a crucial role in mediating anxiety behaviors [56]. Alteration of 5-HT receptor can induce an abnormal brain serotoninergic activity and which prone to increase vulnerability to anxiety. 5-HT1A receptor knockout mice show increased anxiety [57, 58] and in contrast, 5-HT1A receptor overexpressed mice show decreased anxiety [59]. Actually, we have also examined the 5HT5B receptor which is related to social behavior and found that 5HT5B mRNA expression in the prefrontal cortex was not different between the control and arsenic exposed F1 female mice. Recently, it was stated that the forest bathing (walking in the forest) program improve the mood disorders showing increased score for vigor and decreased score for fatigue in the profile of mood states test accompanied with significantly increased serum serotonin level [60]. Moreover, plasma corticosterone level was increased significantly in arsenic-exposed female mice in the present study, and which can cause the release of corticosteroids from the adrenal gland via HPA axis and which may influence postsynatptic 5-HT1A function [61]. Dopamine D2 receptors plays a crucial role in the pathogenesis of anxiety and depression in human and animal studies [62–64]. Moreover, D2 receptors also regulate anxiety-related behavior and elevated plus-maze-associative memory via the interactions of dorsal hippocampus and the nucleus accumbens dopaminergic system [65]. In the present study, dopamine receptor Drd2, mRNAs in the PFC were remarkably reduced in arsenic-exposed female mice.

Regarding immunological changes in the brain, arsenic-exposed F1 female mice showed significantly increased in the expression of apoptosis-related markers (caspase-3 and Bax) and inflammatory molecules (NFκB, IL-1β, COX2). It was suggested that arsenic-induced neuronal apoptosis and neuroinflammatory responses in the PFC. Arsenic triggers apoptosis via free radical generation. It was reported that antioxidants such as ascorbate and α-tocopherol selectively altered the extent of DNA damage by reducing TNF-α level and inhibiting the activation of caspase cascade in male albino rats [66]. In this study, we did not examine the reversible effects of antioxidants. However, we strongly suggest that antioxidant treatment with suitable dose and duration can improve abnormal behavior induced by arsenic exposure via modulation of oxidant-antioxidant balance accompanied with inflammatory markers such as NFκB, IL-1β, and COX2. Thus, dietary vitamin supplement will be helpful for recovery in patient with heavy metal intoxication.

Recently, Gade et al., have reviewed sex-specific neurotoxic effects of heavy metals [67]. They have reported that lead exposure is susceptible to male than female while mercury exposure is susceptible to boy than girl. On the other hand, manganese exposure is susceptible to girl than boy while cadmium exposure is susceptible to female than male. For Arsenic exposure, very limited evidence experimentally and epidemiologically. The factors influencing the sex-specific neurotoxic effects are body mass, nutritional status, socioeconomic status, timing of exposure, genetic background, molecular, hormonal, epigenetic mechanism. Understanding of the sex-specific effects of environmental heavy metal neurotoxicity would be helpful in the development of systematic approach in risk assessment and novel approach in preventive and therapeutic measures.

## Conclusion

The present study indicates that developmental arsenic exposure may induce anxiety-like behavior in animal models via dysregulation of neurotransmitters, impaired neurogenesis and neuroinflammation. Therefore, our study would be helpful to understand the mechanism of arsenic-induced mood disorder following gestational exposure. Further study is needed to explore the transgenerational effects of gestational arsenic exposure in both male and female childhood and young adulthood.

## Abbreviations

F1: first generation
F2: second generation
NaAsO_2_: sodium arsenite
5-HT1A: 5-hydroxytryptamine (serotonin) receptor 1A
Drd2: dopamine receptor D2
BDNF: brain-derived neurotrophic factor
COX-2: cyclooxygenase 2
HO1: heme oxygenase 1
NFκB: nuclear factor kappa B
*IL-1β*: interleukin 1β
Bax: nuclear factor kappa-light-chain-enhancer of activated B cells
HPA: hypothalamic-pituitary-adrenal
PFC: prefrontal cortex

## Declarations

### Availability of data and materials

The data of current study are available from the corresponding author on reasonable request.
